# Multicentric Osteolysis Nodulosis Arthropathy Syndrome Simulating Juvenile Idiopathic Arthritis in an Adult Female: A Case Report and a Literature Review

**DOI:** 10.7759/cureus.45152

**Published:** 2023-09-13

**Authors:** Syed K Imam, Dhekra Alnaqeb, Mohammad Bedaiwi, Ebtissal M Khouj

**Affiliations:** 1 Internal Medicine Department, Sultan Bin Abdulaziz Humanitarian City, Riyadh, SAU; 2 Internal Medicine Department, College of Medicine, King Saud University, Riyadh, SAU; 3 Translational Genomics Department, Centre for Genomic Medicine, King Faisal Specialist Hospital and Research Centre, Riyadh, SAU

**Keywords:** juvenile idiopathic arthritis, autosomal recessive, skeletal dysplasia, matrix metalloproteinases, torg syndrome, winchester syndrome, online mendelian inheritance in man (omim), multicentric osteolysis nodulosis arthropathy

## Abstract

Multicentric osteolysis, nodulosis, and arthropathy (MONA) syndrome is one of the rare genetic skeletal dysplasias, inherited as an autosomal recessive disorder, which predominantly involves carpal and tarsal bones with characteristic osteolytic lesions and can be misdiagnosed as juvenile idiopathic arthritis or rheumatoid arthritis. MONA syndrome includes diseases involving two genes: the matrix metalloproteinase 2 (MMP2) gene and matrix metalloproteinase 14 (MMP14). Both genes are assumed to cause phenotype variants of the same disease. Older patients may manifest some arthritic features, especially in the wrist, and minute pathological fractures can occur as well. These patients may be misdiagnosed as inflammatory arthritis and physicians might prescribe corticosteroid and disease-modifying immunosuppressive agents. Therefore, physicians should carefully evaluate genetic skeletal dysplasia to make a correct diagnosis and avoid unnecessary pharmacological intervention.

We report a case of MONA syndrome in an adult female who came to our facility for an intensive rehabilitation program.

## Introduction

The multicentric osteolysis disorders are inherited group of disorders of the bone characterized by osteopenia, osteoporosis, and progressive bone and joint destruction that lead to skeletal abnormalities with functional impairment and are associated with extra-skeletal features such as congenital cardiac diseases, subcutaneous nodules, dental anomalies, and gum hypertrophy. The International Skeletal Dysplasia Society classifies it into four major groups [1] and our case fits into the first category involving multicentric hands and feet according to this classification.

The development of tissues and organs depends upon extracellular matrix (ECM) breakdown and its turnover is carried out by specialized proteinases, matrix metalloproteinases (MMPs), a family of zinc- and calcium-dependent endopeptidases. There are two types of genes implicated in disease manifestation, the MMP2 gene and the MMP14 gene. Phenotype variants of the same disease are assumed to be caused by both genes.

MMPs regulate the activity of a critical growth factor, transforming growth factor-beta (TGF-β), and signaling of growth factor mediates the coupling of the reciprocal activities of bone formation and resorption by influencing the maturation of osteocytes and enhancing the activity of osteoclasts. MMP2 encodes a member of the MMPs that degrades matrix proteins. They are important mediators of connective tissue remodeling. MMP2 is involved in the hydrolysis of gelatin and type IV collagens, the major structural component of the basement membrane, as well as elastin, laminin, fibronectin, aggrecan, and fibrillin and is believed to be involved in normal collagen turnover and tumor cell invasiveness. Mutations in the genes encoding these proteins lead to defects in proteolysis of the ECM that result in osteolysis, nodulosis, and arthropathy and provide insights into the roles of MMP 2 and the ECM in the pathophysiology of bone and joint development. MONA syndrome is an autosomal inherited rare primary skeletal dysplasia and predominantly involves carpal and tarsal bones with characteristic osteolytic lesions.

MONA syndrome (OMIM # 259600) is inherited in an autosomal recessive manner and the genetic basis of MONA was first described in 2001 by Martignetti et al. [2]. There are 46 patients with 24 different variants in MMP2 reported by Kroger et al. [3], while Vos et al. reported only four patients with two variants in MMP14 [4]. The MMP2 gene is located on chromosome 16 (p12.2). The characteristic skeletal features of MONA syndrome include progressive deformities of hands and feet associated with carpal and tarsal bone osteolysis and osteopenia. Deformities of the hands and feet might be accompanied by subcutaneous nodules that could lead to arthropathy-like manifestations such as stiffness, pain, and swelling [5]. Extra-skeletal manifestations include facial dysmorphism and oral, cardiac, ophthalmological, and dermatological involvement. There is no treatment for MONA syndrome apart from palliative treatment, and data about long-term prognosis are limited. We report a case of multicentric osteolysis, nodulosis, and arthropathy (MONA) syndrome in an adult female simulating juvenile idiopathic arthritis who came to our facility for an intensive rehabilitation program.

## Case presentation

We report a case of an 18-year-old Saudi female, a university student, who came to our center for an intensive rehabilitation program and was referred to a genetic clinic by a rheumatologist.

She had normal developmental milestones until the age of six years when her parents noticed that she had short stature as compared to other siblings and started to develop contraction deformity of both hands and feet and was taken to a local hospital where her evaluation was not conclusive of any documented diagnosis. At the age of 14 years, she complained of bilateral hand and wrist pain and painful swelling in both knees and traveled to Egypt for further evaluation where she was clinically diagnosed with juvenile idiopathic arthritis and methotrexate was prescribed and she took it for two years and quit it by herself due to inconclusive response. She had a history of trauma to her right hand that resulted in a distal radial fracture.

**Figure 1:**
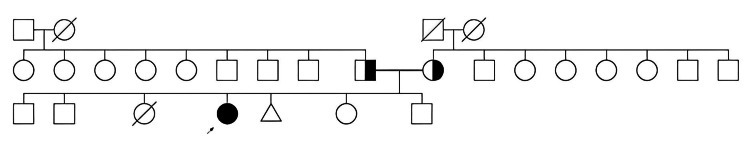
Family pedigree

She was born by normal vaginal delivery to a consanguineous couple and her delivery was uneventful. Her parents and siblings were free of skeletal abnormalities (see Figure 1, family pedigree). She had normal and regular menstrual cycles and once-weekly vitamin D3 was the only medication that she had been taking regularly.

On examination, she was ambulatory and had normal intelligence. Her height was 148 cm and body mass index (BMI) was 25.5 kg/m^2^ and her vital signs were normal. She had dysmorphic facial features, frontal bossing, a depressed nasal bridge, and a bullous nose. Other notable physical characteristics were a subcutaneous nodule on the right plantar surface, black discoloration of the tip of the tongue, a high-arched palate, and teeth malalignment. Hand examination revealed a limited passive and active range of motion around both wrists in extension. Metacarpophalangeal joints were flexed and deformed bilaterally. The right elbow was in valgus position with limited supination. The feet were flat with tender navicular bones bilaterally. Knee examination showed limited flexion mainly on the right side. Chest, cardiovascular, and abdominal examinations were unremarkable. Her higher mental as well as sensory and motor functions were normal. The breast examination was suggestive of Tanner’s stage 2 development.

**Figure 2:**
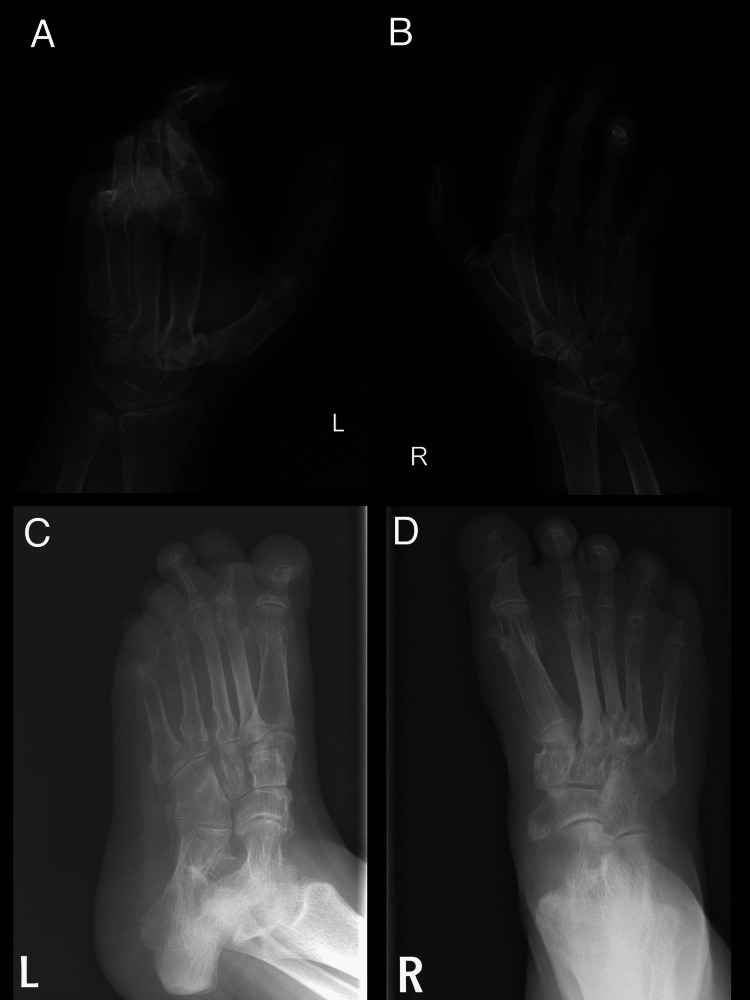
Radiographs of both hands and feet Radiograph of hands: A and B show marked osteopenia, flexion deformity of the interphalangeal joints more at the left hand, bony resorption of the distal part of fifth metacarpal bones and proximal part of middle phalanx of little fingers, Radiograph of feet: C and D show bilateral flat feet with marked osteopenia, destruction of interphalangeal joints of the big toes, fused proximal interphalangeal joints of the remaining toes, and increased bone resorption seen at distal tufts of proximal phalanges of left fourth and fifth toes.

Her complete blood count (CBC), calcium, liver, renal, thyroid, gonadotrophins, and estradiol were normal and low vitamin 25-OH D was the only abnormal laboratory finding. The electrocardiogram showed normal sinus rhythm and the echocardiogram was suggestive of mitral valve prolapse. Radiographs of both hands showed marked osteopenia, flexion deformity, and resorption (Figure 2A, 2B). Radiographs of feet showed bilateral flat feet with marked osteopenia, bony destruction, and resorption (Figure 2C, 2D).

Radiographs of both knees were normal. Dual-energy X-ray absorptiometry (DEXA) scan showed normal bone density of the right femoral neck and osteopenia of the left femoral neck and lumbar spine. The nerve conduction study was normal, and the ultrasound pelvis showed normal ovaries and uterus.

For genetic study, written informed consent of the patient was obtained as per the guidelines of the Institutional Review Board. The blood sample was taken and sequencing of all exons of the MMP2 gene was done by using Affymetrix Genotyping Microarray Chip. The results showed a novel base variant of NM_004530.6(MMP2_i001): p. (Tyr244*) in the homozygous state. Parents were also tested and have been found to be carriers for the above-mentioned variant.

The diagnosis of MONA was established with characteristic clinical and radiographic findings and confirmed by the identification of the mutation in MMP2 on molecular genetic testing. Patient and parents’ education session was conducted, and genetic counseling was done. She was prescribed oral bisphosphonate, calcium, and cholecalciferol and encouraged to actively participate in the rehabilitation program and advised rheumatology follow-up.

## Discussion

MONA syndrome is a rare primary skeletal dysplasia that results from MMP2 gene mutation and is characterized by progressive osteolysis, arthropathy, and extra-skeletal manifestations such as facial dysmorphism, subcutaneous palmar or plantar nodules, pigmented skin lesions, cardiac defects, and corneal opacities and its onset ranges generally between ages six months to six years and most affected children are apparently normal at birth. MONA is inherited in an autosomal recessive manner and each sibling of an affected individual has a 25% chance of being affected, a 50% chance of being an asymptomatic carrier, and a 25% chance of being unaffected. MONA spectrum includes Winchester syndrome, Torg osteolysis syndrome, nodulosis arthropathy osteolysis (NAO) syndrome, and Torg-Winchester syndrome [6-8]. Some authors include Frank-ter-Haar syndrome which is caused by homozygous mutation in the TKS4 gene among the MONA syndrome spectrum disorder [9]. Winchester syndrome (OMIM # 277950) has autosomal recessive inheritance and the gene is located on chromosome 14q11 that encodes MMP14. Homozygous mutation in MMp14 results in disease manifestation, which is characterized by severe osteolysis and generalized osteoporosis; however, subcutaneous nodules are absent. Torg syndrome (OMIM # 259600) is characterized by the same clinical features of MONA; however, osteolysis is limited to the hands and feet and is accompanied by the widening of the metacarpal and metatarsal bones. NAO syndrome (OMIM # 259600) causes massive osteolysis and more severe osteoporosis, painful subcutaneous nodules, and hirsutism. MONA can also be misdiagnosed as juvenile idiopathic arthritis or other skeletal dysplasias.

MONA should be differentiated from other skeletal dysplasias, which can be divided into autosomal recessive and dominant inheritances. Autosomal recessive skeletal dysplasias mimicking MONA include juvenile idiopathic arthritis (OMIM # 618795) [10], hyaline fibromatosis syndrome (OMIM # 228600) [11], and Frank-ter-Haar syndrome (OMIM # 249420) [12], and autosomal dominant skeletal dysplasias simulating MONA include multicentric carpal tarsal osteolysis (OMIM # 166300) [13] and familial expansile osteolysis (OMIM # 174810) [14].

Table 1 compares the clinical features of the MMP2 gene variant of our case with a few previously reported MMP2 gene variants obtained through the literature search, and reported cases mentioned in the table were from Saudi Arabia, Turkey, Egypt, Iran, India, and North America and the first reported case of MMP2 gene variant was described in Saudi Arabian families [2,5]. Positive consanguinity is strongly correlated with the MMP2 gene variants and significantly increases the risk of autosomal recessive disorders. Furthermore, marriages within families are deeply rooted in social norms and trends in the world population mostly residing in the Middle East, West Asia, and North Africa, and almost all cases mentioned in the table were related to these regions [15].

**Table 1:** Comparison of clinical features of the MMP2 gene variant of our case with previously reported MMP2 gene variants NA = information or data not available; DEXA = dual-energy X-ray absorptiometry; MMP2, matrix metalloproteinase 2.

Features	Current case	Al Aqeel et al., 2000 [5]	Zankl et al., 2007 [8]	Tuysuz et al., 2009 [16]	Shakiba and Alaei, 2023 [17]	Elsebaie et al., 2021 [18]	Alazhary and Al Agha, 2017 [19]	Ekbote et al., 2014 [20]
Number	1	2	1	2	1	2	1	1
Gender	Female	Male/Female	Female	Male/Female	Female	Males	Male	Female
Age	18 years	4 years/20 years	13 years	6 years/4 years	5 years and 9 months	6.5 years/4 years	7 years	3 years and 5 months
Age of onset	6 years	First year of life	8 months	6 months/3 months	NA	2 years	4 years	18 months
Consanguinity	Positive	Positive	Positive	Positive	Positive	Positive	Positive	Negative
Short stature	Yes	No	No	No	No	No	Yes	Yes
Deformities	Yes	Yes	Yes	Yes	Yes	Yes	Yes	Yes
Pain	Yes	Yes	Yes	Yes	Yes	Yes	No	No
Swelling	Yes	Yes	Yes	Yes	No	Yes	Yes	Yes
Stiffness	No	Yes	Yes	No	Yes	Yes	Yes	Yes
Facial dysmorphism	Yes	Yes	Yes	Yes	Yes	Yes	No	Yes
High-arched palate	Present	Absent	Present	Present (female)	Absent	Absent	Absent	Absent
Gum hypertrophy	Absent	Absent	Absent	Present	Present	Absent	Present	Absent
Dental anomaly	Present	Absent	Absent	Absent	Present	Present	Present	Absent
Ophthalmic features	Absent	Present	Present	Present	Absent	Present	Absent	Absent
Mental retardation	No	Yes	No	No	NA	No	NA	NA
Subcutaneous nodules	Present	Present	Present	Present	Present	Absent	Present	Present
Hirsutism/hypertrichosis	Absent	Present	NA	Present (male)	Present	Absent	Absent	Present
Cardiac anomaly	Present	Absent	Absent	Present	Present	Present	Absent	Absent
Osteoporosis	No	NA	NA	Yes (female)	No	NA	Yes	NA
Osteopenia	Yes	Yes	Yes	Yes	Yes	Yes	Yes	Yes
Metacarpophalangeal deformity	Yes	Yes	Yes	Yes	Yes	Yes	Yes	Yes
Osteolysis	Yes	Yes	Yes	Yes	Yes	Yes	Yes	Yes
Fractures	No	No	Yes	No	No	Yes	No	No
DEXA scan Z-score	Femoral -0.9 to -1.2, lumbar -1.3	NA	NA	–2.3 (male)	Normal	NA	Total body -4, lumbar -2.4	NA

MMP2 gene mutation is characterized by variable onset of clinical presentation, and in most cases, symptoms began at the age of four years or less; however, in the current case report, the onset of disease and diagnosis were late as compared to other reported cases. Clinical Presentation and skeletal involvement in the current case were similar to other cases but short stature, lack of joint stiffness, and high-arched palate were not seen in the majority of other cases, and severity and nature of joint involvement were comparable among patients presenting with the same mutation. Mental retardation was only reported in Al Aqeel et al. 2000 [5] and it seems that the intelligence level generally remains normal in the MMP2 gene mutation. The link between MMP2 gene mutation and cardiac pathology was first described by Tuysuz et al. [16] in a Turkish family and literature reported many cases of congenital cardiac anomalies linked to MMP2 gene mutation from Middle Eastern regions including current cases [16-18]. Bone resorption, osteopenia, osteolysis, and hand deformities were the characteristic findings in all cases and very few cases showed fracture incidence [8,17]. Clinical and radiological findings are helpful in providing better insight and a prospective understanding of the physiologic role of the MMP 2 gene in human physiology, and molecular findings may clarify which substrates and MMP2 activities are critical in bone, joint, and cardiac development.

There is no specific treatment recommended to treat MONA syndrome. One of the reported cases responded well to oral prednisolone and methotrexate with the improvement of joint pain, contracture, and nodulosis with normal growth while osteolysis did not improve [5], and in another case, corticosteroid resulted in improvement of painful swelling and nodules [16]. The treatment of osteopenia and osteoporosis using bisphosphonates increases bone mineral density and decreases pain but has no impact on osteolysis [5,17,19,20]. Overall, no improvement was observed in osteolysis, skeletal deformities, or delay in disease progression after using immunosuppressive treatment including methotrexate, etanercept, intravenous methylprednisolone, and oral corticosteroids [8,16,18,19].

## Conclusions

The treatment of MONA syndrome is supportive, and little is known about the long-term prognosis apart from the tendency to progression of skeletal features. Therefore, it is prudent to correctly identify MONA syndrome with other closely related skeletal disorders so that inappropriate medical intervention can be avoided, and suitable intervention can be employed. Bisphosphonates should be considered in osteopenia and osteoporosis and the use of disease-modifying antirheumatic drugs should be avoided. Consanguinity is an important risk factor for MONA syndrome, which is of high interest and concern in Saudi Arabia. Preconception and premarital genetic counseling on consanguinity should be part of the training of healthcare providers particularly in highly consanguineous populations. Molecular analysis is also recommended to aid in developing crucial molecules for targeted therapy. Other future treatment includes targeted enzyme therapy with MMP-2 as MMP 2 deficiency is found in these patients, or the use of TGF-β as MMP 2 activates TGF-β.

Patients with MONA syndrome should get multidisciplinary care and patients with skeletal deformities should undergo a rehabilitation program under a physiatrist, and an annual assessment by a rheumatologist and an orthopedic surgeon. We recommend careful cardiology evaluation for congenital heart disease, as it may be the first presentation of a genetic multisystem disorder, and recommend early screening of osteoporosis in patients with MONA syndrome to allow early pharmacological intervention, improve bone health, and minimize fracture incidence.

## References

[1] Hall CM (2002). International nosology and classification of constitutional disorders of bone (2001). Am J Med Genet.

[2] Martignetti JA, Aqeel AA, Sewairi WA (2001). Mutation of the matrix metalloproteinase 2 gene (MMP2) causes a multicentric osteolysis and arthritis syndrome. Nat Genet.

[3] Kröger L, Löppönen T, Ala-Kokko L, Kröger H, Jauhonen HM, Lehti K, Jääskeläinen J (2019). A novel mutation in the matrix metallopeptidase 2 coding gene associated with intrafamilial variability of multicentric osteolysis, nodulosis, and arthropathy. Mol Genet Genomic Med.

[4] de Vos IJ, Wong AS, Welting TJ, Coull BJ, van Steensel MA (2019). Multicentric osteolytic syndromes represent a phenotypic spectrum defined by defective collagen remodeling. Am J Med Genet A.

[5] Al Aqeel A, Al Sewairi W, Edress B, Gorlin RJ, Desnick RJ, Martignetti JA (2000). Inherited multicentric osteolysis with arthritis: a variant resembling Torg syndrome in a Saudi family. Am J Med Genet.

[6] Winchester P, Grossman H, Lim WN, Danes BS (1969). A new acid mucopolysaccharidosis with skeletal deformities simulating rheumatoid arthritis. Am J Roentgenol Radium Ther Nucl Med.

[7] Bhavani GS, Shah H, Shukla A (2016). Clinical and mutation profile of multicentric osteolysis nodulosis and arthropathy. Am J Med Genet A.

[8] Zankl A, Pachman L, Poznanski A (2007). Torg syndrome is caused by inactivating mutations in MMP2 and is allelic to NAO and Winchester syndrome. J Bone Miner Res.

[9] Iqbal Z, Cejudo-Martin P, de Brouwer A (2010). Disruption of the podosome adaptor protein TKS4 (SH3PXD2B) causes the skeletal dysplasia, eye, and cardiac abnormalities of Frank-Ter Haar Syndrome. Am J Hum Genet.

[10] Ostring GT, Singh-Grewal D (2013). Juvenile idiopathic arthritis in the new world of biologics. J Paediatr Child Health.

[11] Casas-Alba D, Martínez-Monseny A, Pino-Ramírez RM (2018). Hyaline fibromatosis syndrome: clinical update and phenotype-genotype correlations. Hum Mutat.

[12] Maas SM, Kayserili H, Lam J, Apak MY, Hennekam RC (2004). Further delineation of Frank-ter Haar syndrome. Am J Med Genet A.

[13] Faber MR, Verlaak R, Fiselier TJ, Hamel BC, Franssen MJ, Gerrits GP (2004). Inherited multicentric osteolysis with carpal-tarsal localisation mimicking juvenile idiopathic arthritis. Eur J Pediatr.

[14] Hughes AE, Ralston SH, Marken J (2000). Mutations in TNFRSF11A, affecting the signal peptide of RANK, cause familial expansile osteolysis. Nat Genet.

[15] Bittles AH, Black ML (2010). Evolution in health and medicine Sackler colloquium: consanguinity, human evolution, and complex diseases. Proc Natl Acad Sci U S A.

[16] Tuysuz B, Mosig R, Altun G, Sancak S, Glucksman MJ, Martignetti JA (2009). A novel matrix metalloproteinase 2 (MMP2) terminal hemopexin domain mutation in a family with multicentric osteolysis with nodulosis and arthritis with cardiac defects. Eur J Hum Genet.

[17] Shakiba M, Alaei F (2023). A novel gene mutation for multicentric osteolysis nodulosis and arthropathy: case report and review of literature. Heliyon.

[18] Elsebaie H, Mansour MA, Elsayed SM, Mahmoud S, El-Sobky TA (2021). Multicentric Osteolysis, Nodulosis, and Arthropathy in two unrelated children with matrix metalloproteinase 2 variants: genetic-skeletal correlations. Bone Rep.

[19] Elazhary H, Al Agha A (2017). Multifocal Osteolysis, Nodulosis, and Arthropathy in a Saudi boy with osteoporosis and short stature. Int J Med Health Res.

[20] Ekbote AV, Danda S, Zankl A, Mandal K, Maguire T, Ungerer K (2014). Patient with mutation in the matrix metalloproteinase 2 (MMP2) gene - a case report and review of the literature. J Clin Res Pediatr Endocrinol.

